# SpliceVarDB: A comprehensive database of experimentally validated human splicing variants

**DOI:** 10.1016/j.ajhg.2024.08.002

**Published:** 2024-09-02

**Authors:** Patricia J. Sullivan, Julian M.W. Quinn, Weilin Wu, Mark Pinese, Mark J. Cowley

**Affiliations:** 1 Children’s Cancer Institute, Lowy Cancer Research Centre, UNSW Sydney, Sydney, NSW, Australia; 2 School of Clinical Medicine, UNSW Medicine & Health, UNSW Sydney, Sydney, NSW, Australia; 3 UNSW Centre for Childhood Cancer Research, UNSW Sydney, Sydney, NSW, Australia

**Keywords:** splicing, variant interpretation, splice region, splice site, splicing regulatory element, genomics, whole-genome sequencing, transcriptomics, pathogenic, canonical splice site

## Abstract

Variants that alter gene splicing are estimated to comprise up to a third of all disease-causing variants, yet they are hard to predict from DNA sequencing data alone. To overcome this, many groups are incorporating RNA-based analyses, which are resource intensive, particularly for diagnostic laboratories. There are thousands of functionally validated variants that induce mis-splicing; however, this information is not consolidated, and they are under-represented in ClinVar, which presents a barrier to variant interpretation and can result in duplication of validation efforts. To address this issue, we developed SpliceVarDB, an online database consolidating over 50,000 variants assayed for their effects on splicing in over 8,000 human genes. We evaluated over 500 published data sources and established a spliceogenicity scale to standardize, harmonize, and consolidate variant validation data generated by a range of experimental protocols. According to the strength of their supporting evidence, variants were classified as “splice-altering” (∼25%), “not splice-altering” (∼25%), and “low-frequency splice-altering” (∼50%), which correspond to weak or indeterminate evidence of spliceogenicity. Importantly, 55% of the splice-altering variants in SpliceVarDB are outside the canonical splice sites (5.6% are deep intronic). These variants can support the variant curation diagnostic pathway and can be used to provide the high-quality data necessary to develop more accurate *in silico* splicing predictors. The variants are accessible through an online platform, SpliceVarDB, with additional features for visualization, variant information, *in silico* predictions, and validation metrics. SpliceVarDB is a very large collection of splice-altering variants and is available at https://splicevardb.org.

## Teaser

Predicting whether a genetic variant will affect mRNA splicing is challenging. With SpliceVarDB we have consolidated and harmonized experimental splicing evidence for over 50,000 genetic variants from over 8,000 genes, half of which affect noncanonical splice sites. SpliceVarDB excels as a variant curation resource and for training better prediction algorithms.

## Introduction

Next-generation sequencing has successfully provided diagnoses for many rare genetic diseases and is becoming a cost-effective approach in many clinical areas. However, despite the increasingly common use of whole-genome sequencing (WGS), the most comprehensive DNA sequencing method to date, diagnosis rates for rare genetic diseases remain around 50%.^1^^,^^2^^,^^3^ Using RNA sequencing (RNA-seq) data in addition to WGS has been shown to increase diagnosis rates by up to an additional 35%^4^^,^^5^^,^^6^; contributing to this is the identification of variants that cause mis-splicing. Splice-altering variants affect pre-mRNA splicing, resulting in altered structure, function, and regulation of their translated protein products.^7^ Although it is usually straightforward to predict the likely functional consequence of a missense or nonsense variant on a transcript sequence, predicting whether a variant affects splicing (and what that effect is) can be more challenging since it may disrupt or create a large number of splicing motifs. Many *in silico* predictors can provide valuable clues for predicting splicing alterations, but in general, they lack the accuracy required to avoid the need for experimental validation in a diagnostic setting.^8^ Indeed, the American College of Medical Genetics and Genomics (ACMG) guidelines do not recommend declaring pathogenic or likely pathogenic status from *in silico* predictions alone.^9^

Experimental validation is thus commonly required to determine the potential clinical significance of predicted splice-altering variants. However, in practice, this constitutes a significant barrier to diagnosis since laboratory validation requires time, expertise, and expensive clinical diagnostic services, without which the variants often remain classified as variants of uncertain significance (VUSs). Splicing validation methods such as RNA-seq and RT-PCR analysis require access to an affected tissue of interest, which may not be available, and nonsense-mediated mRNA decay in the tissues can also mask splicing alterations by degrading the mutation-carrying allele.^7^ Alternatively, minigene assays can determine the effects of knocked-in variants on a size-limited gene product, typically using the immortalized human embryonic kidney cell line HEK293T.^10^ Recent innovations allow these to be performed at scale with massively parallel reporter assays (MPRAs) that evaluate the effects of multiple variants in cultured cell lines.^11^ Although many variant validations by this method have been published, it is technically challenging and not yet feasible for large-scale non-contiguous assays of variants of interest.^12^^,^^13^ Given these technical difficulties in validating putative splice-altering variants, which can be crucial for clinical care, novel approaches are needed.

Information about splice-altering variants is dispersed across many research reports and online resources, making retrieval difficult and time consuming. This disorganization of information has also led to many duplications of effort; for example, of the 257 splice-altering variants validated by Wai et al.^14^ published in 2020, at least 31 were present in the literature prior to publication, with one (a *BRCA1* variant) published in 1995.^15^ Databases that have collated some splice-altering variants exist but have size, usability, and currency limitations. These databases do not indicate the true scale of variants that have been validated for splice-altering potential since, collectively, they contain only 1,295 variants (DBASS3 *n* = 338,^16^ DBASS5 *n* = 601,^16^ MutSpliceDB *n* = 364,^17^ shared *n* = 8). Furthermore, general variant databases such as ClinVar^18^ do not require variants to be functionally validated, affecting their reliability and utility for analyzing a variant’s effect on splicing. The focus of our study is on variant-induced mis-splicing, although there is value in considering naturally occurring alternative splicing events in variant interpretation. For this purpose, we recommend consulting resources like OncoSplicing^19^ and SpliceVault.^20^

Here, we present SpliceVarDB, a comprehensive database of variants functionally demonstrated to affect (or not affect) splicing. SpliceVarDB aims to accelerate the diagnostic process for individuals with rare genetic diseases by consolidating information about functionally validated splice-altering variants into a central, accessible repository. This online database enables researchers to quickly access and evaluate previously validated variants, reducing the need to validate suspected variants of interest. SpliceVarDB can thus improve the accuracy and efficiency of variant analysis to enhance the quality of clinical care. This large resource also facilitates the development of more accurate machine learning models for *in silico* splicing predictors as it provides the high-quality training data required for machine learning.

## Material and methods

The variant collection was performed using Scopus to identify published studies that performed the functional assessment of variants with putative splice-altering potential. The following search terms were required to be present in the article title, abstract, or keywords: “splic^∗^” and “mutation” or “variant” and “RNA-seq” or “minigene” or “cDNA” or “RT-PCR” or “splicing assay.”

We note that almost 10,000 studies are returned using the above search criteria, and not all papers were screened for inclusion in the initial set of SpliceVarDB variants described here. Newer studies were more likely to be included due to chronological sorting. Studies were selected for inclusion based on manual abstract review followed by manual determination that the study methodology was described sufficiently. Studies were included when they presented results for both the variant and wild type through gel electrophoresis visualization or sequencing to confirm splice alterations. Some variants were excluded based on non-standard wild-type allele presentation such as an unreported cryptic splice site used over the canonical when no variants were present. For papers with many validated variants (≥50), variants were included if the authors defined thresholds for splice-altering status and consistent methodologies used for validation.

After accumulating over 1,000 variants that alter splicing from smaller-scale publications, we altered our search terms to enrich the dataset for more unusual splice-altering variants. A subsequent search focused on identifying publications featuring variants not located at acceptor or donor splice sites to enhance the diversity of SpliceVarDB. Consequently, we incorporated the following search terms to augment our initial search criteria: “deep intronic” or “splicing enhancer” or “splicing silencer” or “branchpoint” or “pseudoexon.”

Studies that contributed a significant number of variants were MFASS,^21^ MaPSy,^12^^,^^22^ SAVNet,^23^ and MiSplice.^24^

The following information was collected or manually determined from each study: the variant location (i.e., genomic location and coordinates defined by the Human Genome Variation Society [HGVS]), the type of validation performed, tissue or cell line of validation, the splicing element altered, and the reported consequence of the variant on the transcript. Here, we utilize the standard splicing definitions of exon skipping, intron retention (where the entire intron is retained), and pseudoexon inclusion. We also employ the accepted terminology of “exon extension,” where part of an intronic sequence is included, and “exon truncation,” where part of an exonic sequence is excluded from the transcript. For studies that did not report the genomic coordinates, we determined the coordinates using TransVar.^25^ Genomic coordinates for variants reported using intervening sequence nomenclature were determined by examination of RefSeq^26^ transcripts. SpliceVarDB supports hg19 and hg38 genome builds, and variants were converted between builds using *liftOver* and chain files provided by the UCSC Genome Browser.^27^ Variants were left normalized, and the reference bases were checked for both reference genomes using *bcftools norm*^28^ to ensure alignment standardization.

We classified the variants into three categories: splice-altering, low-frequency splice-altering, and “normal” splicing, using the criteria established in Table 1. The thresholds used are predominantly based on those defined by the study itself. However, most studies only defined criteria for splice-altering variants and did not define criteria for variants that resulted in normal splicing; therefore, we implemented stringent thresholds to define the normal category to ensure a high-quality set of control variants. Those that did not meet these criteria were classified as low-frequency splice-altering variants with a wide range of sub-optimal scores; these variants are still included in SpliceVarDB, and while they could be splice-altering, they are not recommended for use in training *in silico* prediction models. In situations where a variant was validated multiple times, if at least one validation returned splice-altering and another returned normal, the “conflicting” category was applied. If low-frequency splice-altering was observed in combination with splice-altering or normal, the low-frequency splice-altering category was applied.

**Table 1:** The criteria and the threshold values used to categorize variants according to splice-alteration severity

**Thresholds for inclusion in category**					
Dataset	Value (x)	Threshold reported by study	Splice-altering	Low-frequency splice-altering	Normal

Literature	transcript abundance change	N/A	x ≥ 10%	N/A	x < 3%
SAVNet	RNA reads AND/OR Bayes factor	N/A x ≥ 3	x ≥ 5 OR x ≥ 10	1 ≤ x < 5 AND 3 ≤ x < 10	N/A
MiSplice	RNA Reads AND JAF	x ≥ 5 AND x ≥ 0.05	x ≥ 5 AND x ≥ 0.05	N/A	N/A
MFASS	Δ Inclusion index	x ≤ −0.50	x ≤ −0.50	0.03 ≤ |x| < 0.5	|x| < 0.03
MaPSY	Allelic ratio AND/OR *p* value	|x|— ≥ 1.5^a^ AND x < 0.05	|x|— ≥ 1.5 AND x < 0.05	|x| ≥ 0.1 AND/OR x ≥ 0.05	|x| < 0.1

Values for splice-altering status reported by each original study are included. When multiple values are used for categorizing the variants within a dataset, both criteria (AND) or one criterion (OR) is required to be satisfied. N/A,not applicable; JAF, junction allele frequency; Δ inclusion index, the change in the inclusion index.

a Alternative criteria to the threshold reported by the study were used but not disclosed.

Genes and variant locations were obtained using GENCODE v44.^29^ Splice regions were calculated as specific distances from the closest canonical exon, including 5′ and 3′ untranslated regions (UTRs). HGVS coordinates for intronic variants could be used interchangeably.

The ClinGen dataset consisted of genes with a Gene-Disease Clinical Validity of moderate or higher^30^ (downloaded January 20, 2024). COSMIC encompassed all tier 1 or 2 genes from the Cancer Gene Census^31^ (downloaded May 29, 2024). Gene2Phenotype (G2P) included genes from all panels with at least a moderate confidence level^32^ (downloaded May 29, 2024). Online Mendelian Inheritance in Man (OMIM)^33^ gene lists included only genes with a phenotype mapping method of 3, thus including only genes where the molecular basis for the disorder is known (genemap2.txt file generated May 31, 2022). The ClinGen Clinical Domain for the genes was determined by mapping the expert panel that curated the Gene-Disease Clinical Validity to their ClinGen Clinical Domain Working Groups. HGVS notation, Genome Aggregation Database (gnomAD) v2.1 allele frequency,^34^ and variant effect predictor (VEP)^35^ most severe consequence was obtained for all variants using Ensembl application programming interface (API).^36^ Variant pathogenicity was determined by annotating variants with the clinical significance (CLN_SIG) as determined by ClinVar^18^ (clinvar_20230923.vcf.gz).

To predict the rate of all splice-altering variants in SpliceVarDB that might be classified as pathogenic or likely pathogenic (P/LP) in the future, the upper limit is defined by the proportion that were already classified as P/LP in ClinVar and splice-altering by SpliceVarDB and the lower limit by the same restricted to OMIM disease genes.

Genomic visualization elements of SpliceVarDB use ProteinPaint^37^ and Integrative Genomics Viewer (IGV) visualization.^38^ Variant information is obtained through the myVariant (https://myvariant.info),^39^ myGene (https://mygene.info),^39^ and ClinVar^18^ APIs. Splicing *in silico* scores are calculated for Introme,^40^ Pangolin,^41^ and SpliceAI^42^ using the API for https://spliceailookup.broadinstitute.org, which runs modified versions of Pangolin and SpliceAI.

## Results

**Figure 1:**
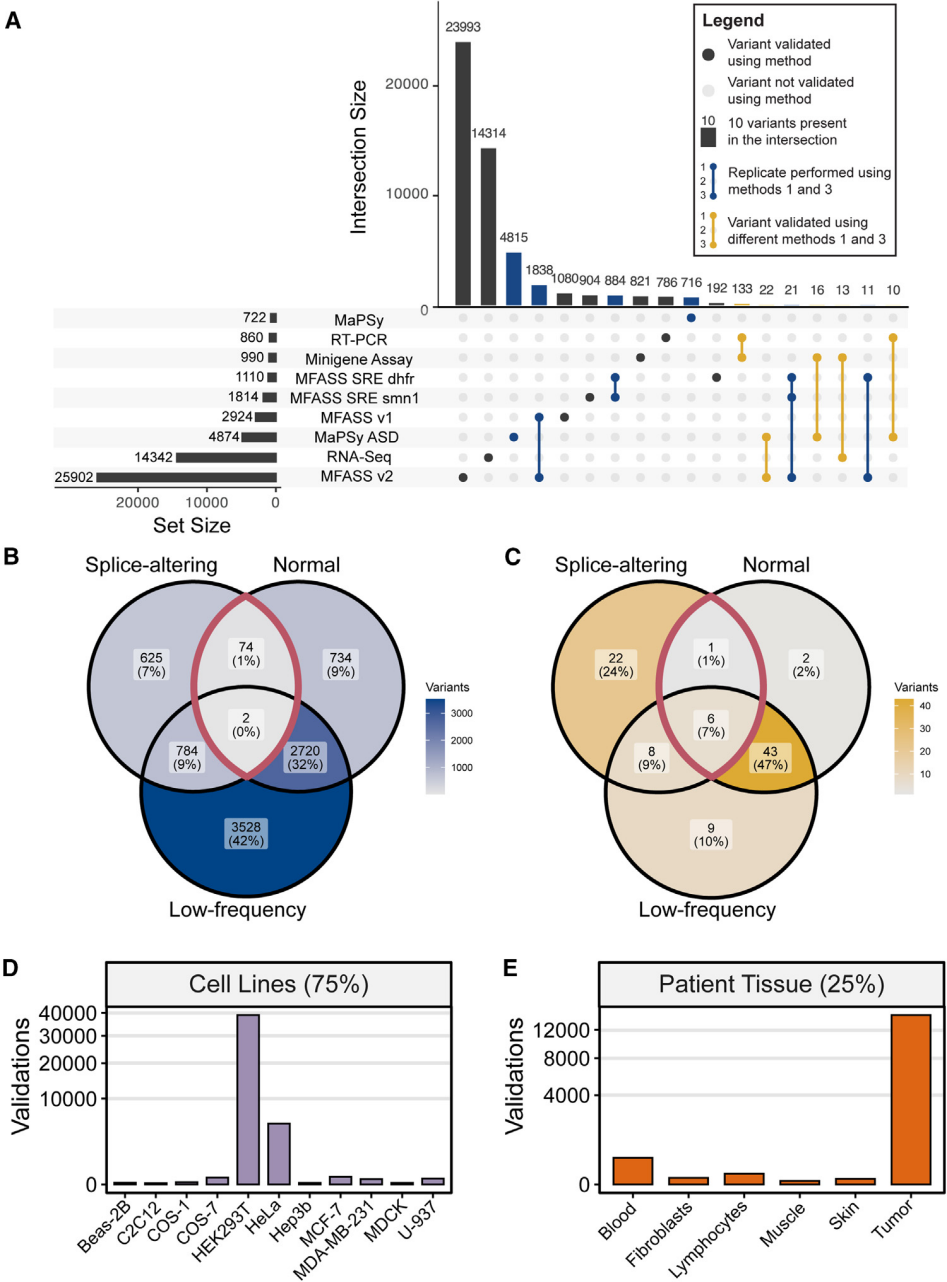
Validation overview for SpliceVarDB variants (A) UpSet^43^ plot of the methods used to validate splice-altering variants with set intersection size and individual set size plotted for the combinations of methods. Intersections in blue demonstrate variants validated with multiple methods, but as replicates within the same study, whereas yellow intersections are variants validated with multiple methods by different studies. (B and C) Venn diagrams of the classification results for (B), variants validated in replicate, and (C), variants validated by multiple studies. Sections outlined in red are classified as conflicting. (D and E) Tissue used for validation for (D), variants assayed using cell lines, or (E), variants validated using clinical tissue samples.

A total of 237 studies were incorporated into SpliceVarDB, reporting between 1 and 28,962 variants each (Table S1). At the time of publication, SpliceVarDB (https://splicevardb.org) contains 50,715 unique variants experimentally assessed for splice-altering potential. Using thresholds defined in Table 1, we recorded 13,673 (27.0%) splice-altering variants, 25,601 low-frequency splice-altering (50.5%) variants, and 11,358 (22.4%) normal variants (Table 2). Of the unique variants identified, 34,530 (68%) were reported in MPRAs (MFASS^21^ and MaPSy^12^^,^^22^), and 14,206 (28%) were variants identified through large-cohort RNA-seq data, such as The Cancer Genome Atlas (https://www.cancer.gov/tcga) (SAVNet^23^ and MiSplice^24^). An additional 2,154 (4.2%) variants validated using various methods were manually compiled from 233 studies (Figure 1A).

**Table 2:** Summary of variants in SpliceVarDB

**Classification**				
Dataset	Total	Splice-altering	Low-frequency splice-altering	Normal

Literature	2,154	1,314	0	836
SAVNet	13,864	10,460	3,404	0
MiSplice	559	559	0	0
MFASS	28,962	1,468	17,357	10,115
MaPSY	5,595	208	4,868	459
Total	50,715	13,673	25,601	11,358

Criteria for classification determination is outlined in Table 1. The table did not include variants classified as conflicting as this classification was applied across multiple validation types.

Of the variants identified, 8,558 variants were validated more than once; 97% were replicates (Figure 1B), and the remaining 3% were reported by multiple studies (Figure 1C). Of the variants validated more than once, 83 were classified as conflicting, meaning that at least one validation determined the variant to be splice-altering whereas another determined the variant to be normal. However, concordance between classifications was generally high, with the conflicting category only applying to 1% of replicates and 8% of variants validated using multiple methods. The validation experiments were primarily conducted with cell lines, accounting for 75% of the experimental validation, compared to 25% that utilized clinical tissue samples (Figures 1D and 1E).

**Figure 2:**
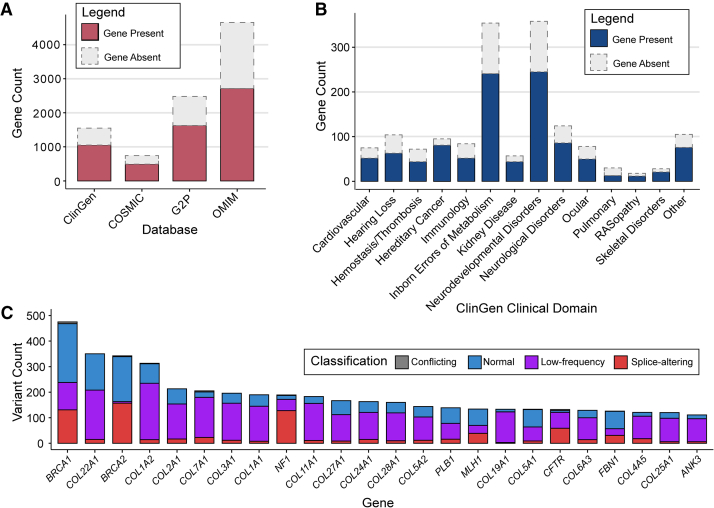
Gene information overview for SpliceVarDB (A) Overlap between genes from online gene databases and SpliceVarDB. Colored bars depict the numbers of genes in the database and SpliceVarDB, whereas dotted bars indicate the full number of genes in the indicated database. (B) Overlap between genes in ClinGen Clinical Domains and SpliceVarDB. Colored bars depict the numbers of genes in the clinical domain and SpliceVarDB, whereas dotted bars indicate the full number of genes in the domain. (C) The variant counts for each gene with greater than 100 functionally validated variants. Variants functionally validated to alter splicing are shown in red, while those that showed no splicing alterations compared to controls are shown in blue, and low-frequency splice-altering variants are in purple.

At the time of publication, SpliceVarDB variants covered 8,362 genes. We referred to established online gene-disease databases to assess the intersection between SpliceVarDB genes and genes implicated in clinical conditions. SpliceVarDB encompasses many of these genes, with coverage ranging from 58.4% in OMIM to 67.8% in ClinGen (Figure 2A). These genes span a broad range of disease categories, as evidenced by their presence across various clinical domains in ClinGen (Figure 2B). Notably, genes in SpliceVarDB most comprehensively represent the hereditary cancer domain at 85%. In contrast, the pulmonary domain had the lowest representation, with only 43% of its genes included. Furthermore, SpliceVarDB includes several extensively validated genes, with 24 genes featuring over 100 functionally validated variants (Figure 2C).

**Figure 3:**
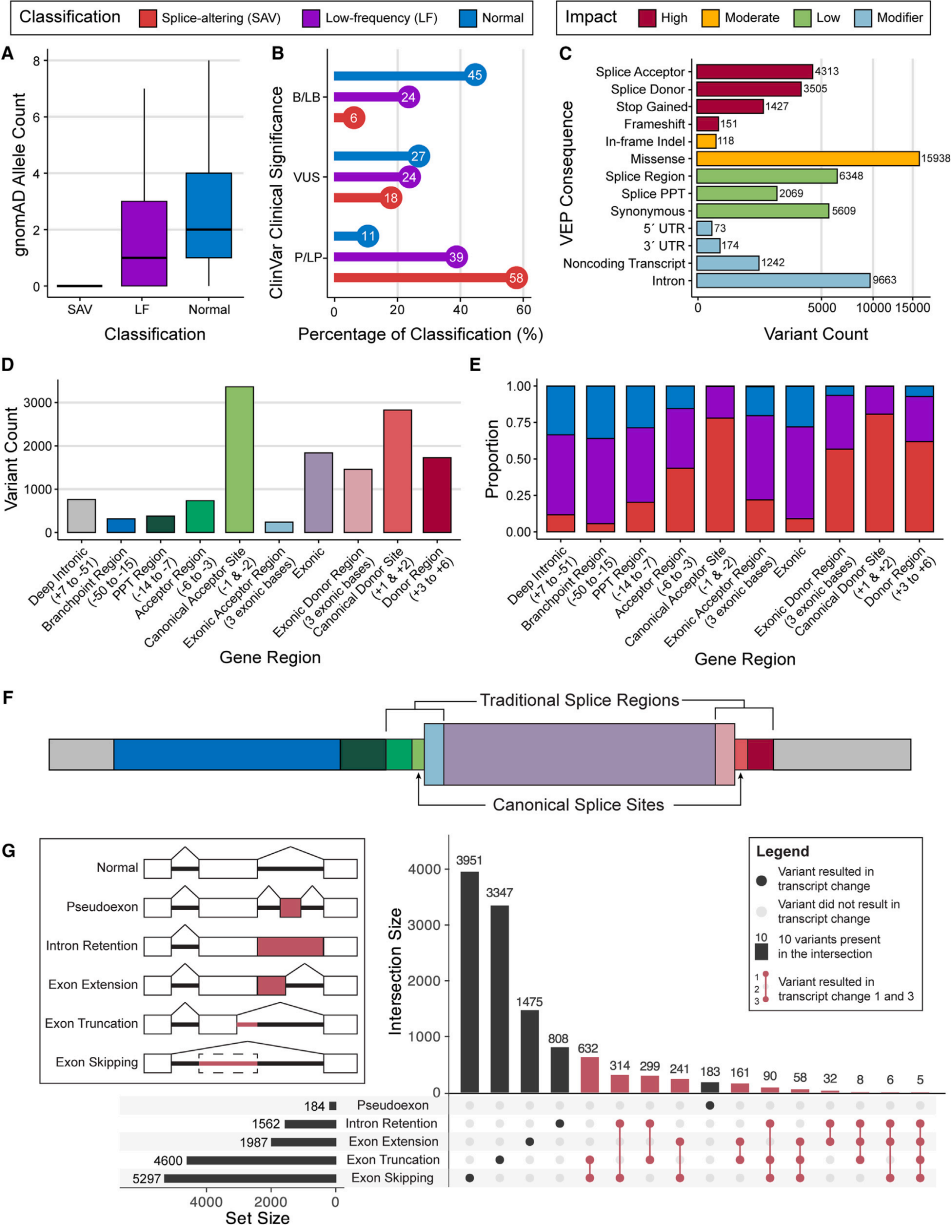
Variant information overview for SpliceVarDB (A) Allele count distribution for each SpliceVarDB classification. (B) Proportion of SpliceVarDB classification’s variants in each ClinVar clinical significance category. Proportion was calculated to include all variants present in ClinVar as the denominator. B/LB, benign/likely benign; VUS, variant of uncertain significance; P/LP, pathogenic/likely pathogenic. (C) Most severe VEP consequence for all variants present in SpliceVarDB. Variants are colored according to the severity of the determined impact. Consequences with less than 50 variants are not shown. (D) Splice-altering variant count for each splicing region as defined in the X axis (distance to the intron-exon boundary in brackets). Colors of the splicing regions are aligned with the feature shown in (F). (E) Splicing classification of the variants in each splicing region (defined in brackets) as represented as a proportion of all variants in the region. Variant color key is as defined in (A). (F) Cartoon depiction of the splicing region locations (with exon shown as the large central rectangle) with colors aligned with the splicing regions shown in the histogram in (D). To show splice-altering variant frequency comparative to size, sections are to scale. (G) UpSet^43^ plot of the transcript changes observed due to the splice-altering variants. Multiple transcript changes can be reported for each variant, represented by the red connected filled-in dots. Diagrams depicting splicing outcome events are shown at the top left, with red lines indicating the exclusion of exonic sequence and red boxes indicating the inclusion of intronic sequence. PPT, polypyrimidine tract.

The variants in SpliceVarDB were generally rare, on average occurring at low frequencies in control datasets. Splice-altering variants were more likely to be absent from gnomAD, whereas variants classified as normal had a median of two alleles present in gnomAD (Figure 3A); however, this is still considered rare, corresponding to an allele frequency of approximately $6\times 10^{-6}$. Exploring the other end of the rarity spectrum, specifically common variants with allele frequencies over 0.01, our analysis identified 240 as normal and 26 as splice-altering.

Most of the splice-altering variants included in SpliceVarDB are not found in ClinVar,^18^ with only 11% having a ClinVar entry; more than half of these were classified in ClinVar as P/LP (58%), with some classified as benign or likely benign (B/LB; 6.4%) (Figure 3B). Notably, splice-altering variants were significantly more likely than normal variants to be classified as P/LP in ClinVar (58% vs. 11%; Fisher’s exact test: *p* < $1\times 10^{-5}$). The low-frequency splice-altering class of variants falls between the splice-altering and normal classes regarding pathogenicity composition (Figure 3B). The variants in SpliceVarDB were analyzed for their most severe consequence as predicted by VEP, returning a high proportion of missense and intronic variants (Figure 3C). Of the splice-altering variants, 64% have high or moderate impact according to VEP, and 70% received a consequence related to splicing. To capture variants that may be deemed pathogenic due to non-splicing mechanisms, analysis of the ClinVar P/LP variants found that 98% of the normal class had a non-splicing consequence of high or moderate impact (e.g., stop gain or missense), compared to 21% of the splice-altering class.

Various factors contribute to determining the pathogenicity of a splice-altering variant. Among these factors, the effect that a variant has on the transcript is critical in assessing its clinical significance since not all splice-altering variants damage the reading frame. Furthermore, the gene that harbors the variant and the frequency of that variant’s occurrence in other individuals (both unaffected individuals and those with similar phenotypes) also play a role in determining its pathogenicity classification. The additional factors used to determine the clinical significance of the variants present in ClinVar, such as patient and family history, are not publicly available. We reasoned that previously classified variants could be used to estimate the number of pathogenic splice-altering variants in SpliceVarDB. We predict that between 2,590 and 7,030 of the splice-altering variants that are not reported in ClinVar will be P/LP (see material and methods). SpliceVarDB may also be useful to refine the classification of variants in ClinVar: those classified as VUSs (18%), with conflicting interpretations of pathogenicity (11%), or unclassified (7%) could potentially be upgraded due to their splice-altering classification (Figure 3B).

Some regions are more likely to harbor splicing-altering variants due to the motif present being essential for splicing, such as the canonical acceptor and donor splice sites. Regions including the latter motifs show a high proportion (approximately 80%) of variants reaching the threshold to be classified as splice altering (Figure 3E). Variants affecting the canonical dinucleotides are generally well-identified, making up 45% of the variants classified as splice altering in SpliceVarDB (Figure 3E). However, these only constitute a small part of the splicing landscape, encompassing four nucleotides per excised intron (Figure 3F). Aside from the acceptor and donor, most splicing motifs do not have a specific distance from the intron-exon boundary in which they must fall; nevertheless, most do have a range of optimal distances. Variants that occur in splicing motifs that do not have a strictly fixed location are less likely to affect splicing than variants occurring in a fixed location motif (Figure 3D). This is evident with the branchpoint and polypyrimidine tract regions: both motifs are essential for the recognition of the acceptor splice site, but only 5.5% and 20% of variants falling in those regions, respectively, are classified as splice-altering (Figure 3E) due to the broad interval into which the motifs may fall while still retaining their function (Figure 3D).

The most common transcript alteration caused by these splice-altering variants was exon skipping (39%), with 14% of variants reported to produce multiple splice-altered transcripts (Figure 3G). This metric is not available for all variants as MPRAs often do not report the effect of the variant on the transcript.

**Figure 4:**
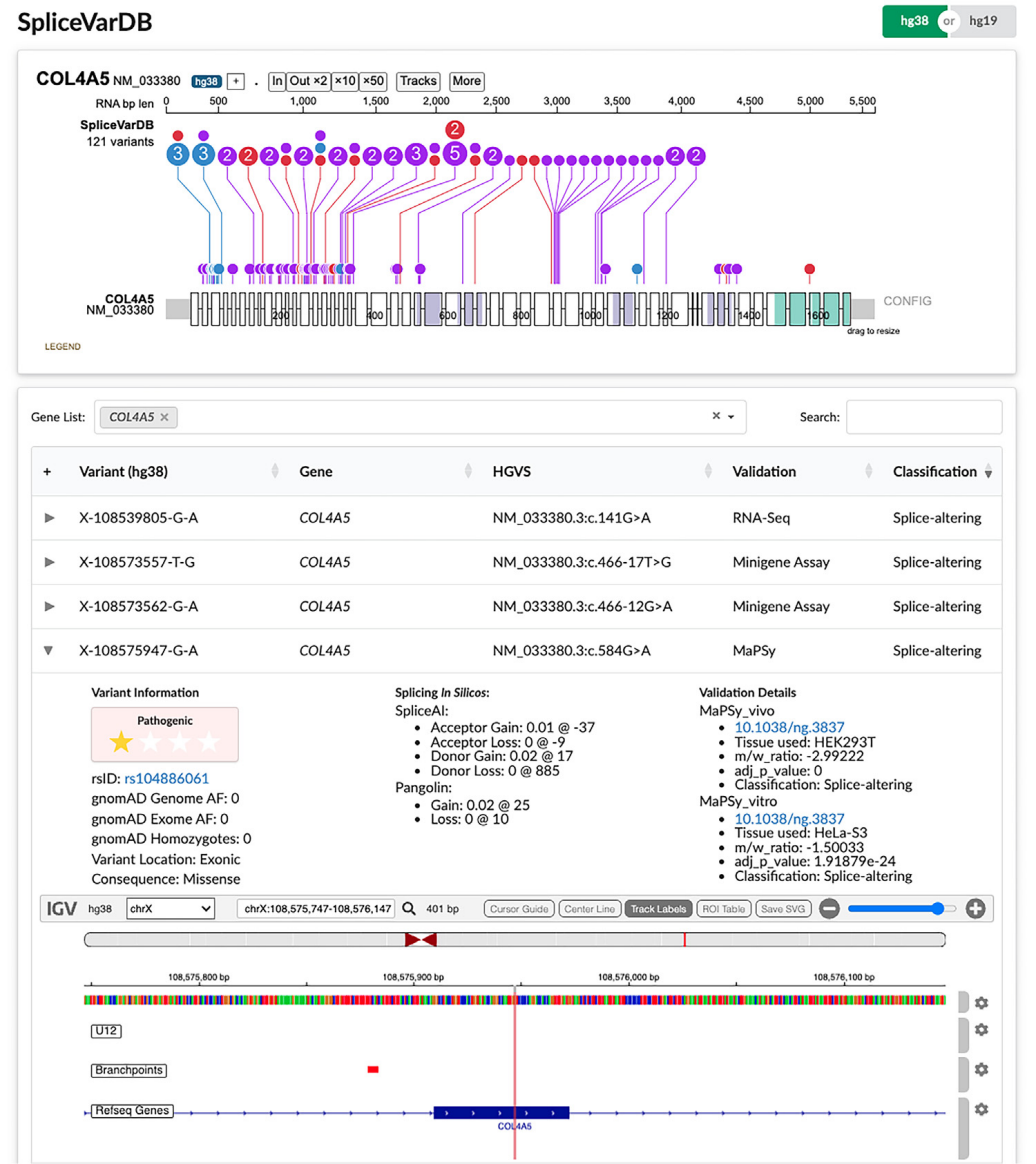
SpliceVarDB online display Lollipop plot showing the variant distribution in *COL4A5* (*n* = 121) generated using ProteinPaint.^37^ Variants are shown with reference to the canonical transcript with exons represented by white boxes (to scale), introns as the space between boxes (not to scale), and the UTRs shown in gray. Variants proven to alter splicing are shown in red, while those that showed no splicing alterations compared to controls are shown in blue; low-frequency splice-altering variants are shown in purple, and variants with conflicting interpretations are in gray. Colored regions of the exons represent protein domains. The variants are shown in a searchable table, with each variant entry expandable to show variant information, *in silico* scores, and validation details. The IGV plot displays the variant (highlighted in the color corresponding to the classification) and nearby splicing elements.

Figure 4 shows an example of how SpliceVarDB can be used to visualize and explore variants in a gene of interest; in this case, *COL4A5* (*n* = 121) was examined. SpliceVarDB generates a ProteinPaint^37^ lollipop plot to demonstrate the locational distribution of variants (classified by whether they affect or do not affect splicing) along the canonical transcript. This distributional view can identify splicing hotspot regions, compare variant outcomes at the same location, and show how well covered the gene is regarding splicing validation. The variants that match the search and filtering criteria are displayed in an expandable variant table (Figure 4). Each entry contains further variant information, *in silico* prediction scores, validation details, and an embedded IGV visualization.^38^ The IGV tracks display nearby splicing motifs (both known and predicted) generated using Introme.^40^

SpliceVarDB offers gene search, variant search, and filtering options based on validation method, variant location, and transcript outcome. It also caters for variants proven not to affect splicing, with 11,358 negative variants in the database. Variants can be uploaded to SpliceVarDB through the “submit variants” function. Researchers can submit published or unpublished variants; the latter enables the capture of variants that are not sufficiently novel for presentation in reports. These variants will be incorporated into SpliceVarDB following manual review.

For registered users, variants and their static annotations can be downloaded from SpliceVarDB in a VCF-like format, which can thus be easily incorporated into variant annotation pipelines. We also provide an API with endpoints for all information used to populate the web page.

## Discussion

Splice-altering variants are an important class of pathogenic variants that can be overlooked due to difficulties associated with predicting and validating their effect on splicing. To address this issue, SpliceVarDB enables access to thousands of variants already experimentally assessed for splice-altering potential, providing researchers with information crucial to variant curation.

The three-tier system for classifying splice-altering variants used by SpliceVarDB harmonizes data from multiple studies and allows researchers to interpret variants at a glance. This classification is based on a threshold set for each functional assay performed (defined in Table 1). Most studies only defined criteria for splice-altering variants and did not define criteria for variants that resulted in normal splicing; therefore, we implemented stringent thresholds to define the normal splicing category to ensure a high-quality set of control variants. Variants that fell between the normal and splice-altering classifications were placed into a low-frequency splice-altering category to ensure they were still captured in SpliceVarDB and available for analysis and interpretation at the researcher’s discretion. This category is likely to contain hypomorphic (partial loss of function) variants, which often contribute to disease,^44^ as well as variants that cause a higher incidence of altered splicing in a different tissue to the one sampled.^45^ Validation results are accompanied by information on the tissue or cell line utilized whenever feasible. Various reports indicate discrepancies in splice-altering variant validation across tissues,^5^^,^^46^^,^^47^ and thus we recommend users assess the relevance of the tissue or assay in relation to a variant of interest.^47^ The tissue used for validation is especially important for interpreting variants in alternatively spliced exons or affecting splicing regulatory elements due to their tissue-specific nature.^48^

SpliceVarDB is not an exhaustive resource of all variants experimentally tested for splice-altering potential. Some variants may have been missed; for example, the publications that were returned using the search terms were too numerous to be comprehensively analyzed. In addition, some publications may also have been excluded due to their lacking a required search term in their article abstract, title, or keywords, which we observed was the case for cohort analysis studies involving (but not centered on) splicing.^49^ SpliceVarDB accepts submissions of published and unpublished variants validated for splice-altering potential as a form of crowdsourcing validation that will help capture variants missed by our literature searches. Smaller-scale variant publications will likely become less prominent as high-throughput analysis methods will be increasingly favored due to their ability to assess variants at scale.^11^ However, RNA results from a tissue of interest will remain a high-quality resource that directly enumerates the splicing outcomes expected in patients. Indeed, as many studies have described the diagnostic yield and variant elucidation benefits gained from performing RNA-seq,^4^^,^^5^^,^^6^^,^^49^ we expect to see further RNA analyses performed. To predict the consequence of splice-altering variants that are not in SpliceVarDB, we recommend considering the consequence of nearby splice-altering variants and/or catalogs of rare cryptic splice junctions like OncoSplicing^19^ and SpliceVault.^20^

We envision SpliceVarDB will also be of considerable use in research areas outside of variant curation. One particular use of high-quality validated results of particular importance is the development of *in silico* splicing prediction tools. These tools are produced by methods such as machine learning that depend critically on the quality of the training data.^50^ Experimentally validated variants, both confidently with and without evidence for altering splicing, provide the high-quality training data necessary for such prediction tools. For example, variants from SpliceVarDB formed the training data for the ensemble machine learning splice predictor Introme.^40^ This dataset enabled us to make Introme, which has half the false positive rate compared to other leading splice-prediction tools.^40^ These variants have also been used as a truth set for comprehensive *in silico* benchmarking of the leading splice-altering prediction tools.^40^ We also envisage SpliceVarDB will reduce the duplication of validation efforts for splice-altering variants, helping to prioritize experimental resources on unvalidated variants of interest to advance knowledge in the field. Splice-altering variants are also emerging as a prime target for personalized therapies^51^ through antisense oligonucleotide (ASO) approaches to counter or correct splicing.^52^ Clearly, a resource such as SpliceVarDB would be significantly useful for selecting candidate variants for such therapy development. User interface elements such as the IGV integration allow rapid visualization of nearby splicing elements and gene architecture, providing useful information in the design of ASOs. Furthermore, ProteinPaint plots can be used to view nearby variants amenable to splicing correction, expanding the utility of these often *n* = 1 gene therapy approaches.^37^

In summary, we have created a resource that details a very large collection of variants validated for splice-altering potential. This is particularly interesting given the relative lack of consolidated information on splice-altering variants, despite their undeniable importance in identifying rare disease cause and understanding cancer behavior. This resource can be easily expanded to accommodate new knowledge and is available with an interactive platform at https://splicevardb.org.

## Data and code availability

The data generated by this study are available at https://splicevardb.org. The browsing of data in the SpliceVarDB website is free to all users. Downloading the SpliceVarDB annotations is free for research use in an academic setting, requires a fee-free license in a private or public diagnostic laboratory, and requires a commercial license in a commercial setting. Additional information is provided in the user registration portal.

The website code is available at https://github.com/CCICB/SpliceVarDB under an AGPLv3 license.

The code used to generate the figures in the paper is available at https://github.com/CCICB/SpliceVarDB.

## Acknowledgments

The authors would like to thank the past and future contributors of variants to SpliceVarDB. P.J.S. was supported by an Australian Government Research Training Program (RTP) scholarship, the Kids Cancer Alliance PhD Top Up scholarship, Petre Foundation PhD Top Up scholarship, and a Fulbright Future scholarship. M.P. is supported by an NHMRC Investigator Grant (#1176265). M.J.C. was supported by an NSW Health Early-Mid Career Fellowship and the Medical Research Future Fund (MRFF) Emerging Priorities and Consumer-Driven Research initiative. This project was supported by grant 1165556 awarded through the 2018 Priority-driven Collaborative Cancer Research Scheme and co-funded by Cancer Australia and My Room. The authors would like to acknowledge Luminesce Alliance – Innovation for Children’s Health for its contribution and support. Luminesce Alliance is a not-for-profit cooperative joint venture between the Sydney Children’s Hospitals Network, the Children’s Medical Research Institute, and the Children’s Cancer Institute. It has been established with the support of the NSW Government to coordinate and integrate pediatric research. Luminesce Alliance is also affiliated with the University of Sydney and the University of New South Wales Sydney.

## Author contributions

P.J.S. conducted the literature review, analyzed the variants, and designed and developed the online platform. W.W. provided back-end website support. P.J.S. and M.J.C. conceptualized the study. P.J.S., J.M.W.Q., M.P., and M.J.C. wrote the paper. M.P. and M.J.C. supervised, and M.J.C. obtained funding for the study. All authors reviewed and approved the manuscript.

## Declaration of interests

The authors declare that they have no competing interests.

## Web resources

SpliceVarDB, https://splicevardb.org
SpliceVarDB API, https://compbio.ccia.org.au/splicevardb-api/doc

## Supplemental information

Table S1. Published studies used in the production of SpliceVarDB
